# Single stranded adeno-associated virus achieves efficient gene transfer to anterior segment in the mouse eye

**DOI:** 10.1371/journal.pone.0182473

**Published:** 2017-08-01

**Authors:** Li Wang, Ru Xiao, Eva Andres-Mateos, Luk H. Vandenberghe

**Affiliations:** 1 Department of Ophthalmology and Visual Science and Eye Research Institute, Eye, and ENT Hospital, Shanghai Medical College, Fudan University, Shanghai, China; 2 Key Laboratory of Myopia, Ministry of Health, Fudan University, Shanghai, China; 3 Shanghai Key Laboratory of Visual Impairment and Restoration, Fudan University, Shanghai, China; 4 Grousbeck Gene Therapy Center, Schepens Eye Research Institute and Massachusetts Eye and Ear Infirmary, Boston, Massachusetts, United States of America; 5 Ocular Genomics Institute, Department of Ophthalmology, Harvard Medical School, Boston, Massachusetts, United States of America; 6 Harvard Stem Cell Institute, Harvard University, Cambridge, Massachusetts, United States of America; Justus Liebig Universitat Giessen, GERMANY

## Abstract

Adeno-associated viruses (AAVs) are used extensively as a gene delivery vehicle for retinal gene therapy, yet its ability to target the anterior segment of the eye, critical to unlocking therapeutic opportunities, is less characterized. Previously, self-complimentary (sc) AAV was shown to be necessary for transduction of the cornea and trabecular meshwork (TM), limiting the size of the gene transfer cassette, likely due to a block in second strand synthesis thought to be required for functional transduction. Here, we evaluated several AAV capsids in a single stranded (ss) genome conformation for their ability to overcome the need for scAAV for targeting corneal endothelium and TM. AAV2, 8, and a recently synthetically developed AAV called Anc80L65 were evaluated *in vitro* and *in vivo* by intracameral injection in mice. Results show that although scAAV2 demonstrated superior infectivity *in vitro* including Human Trabecular meshwork (HTM) immortalized cell lines; Anc80L65 transduced following a single intracameral injection efficiently all components of the mouse anterior segment, including the TM, corneal stroma, and endothelial cells. These results suggest that Anc80L65 is able to overcome the requirement for scAAV genomes to enable TM and corneal targeting, expanding the potential experimental and therapeutic use of AAV gene transfer in the anterior segment of the eye.

## Introduction

The adeno-associated virus (AAV) is small single-stranded DNA virus permissive in humans. As a recombinant vector, AAVs are able to transduce non-dividing cells resulting in long term transgene expression[1] and therefore have found utility in the field of gene therapy. AAV gene therapy for a form of inherited retinal degeneration and hemophilia and multiple preclinical efforts, has identified key properties for a vector system in relation to its tissue and disease target that enable successful application; high tissue tropism, sufficient cargo capacity, adequately robust transgene expression, a minimal level of integration of host genome, and the absence of immune responses and toxicity[2].

AAVs vectors have been used widely in gene therapy research and clinical trials for ocular diseases, due to important advantages of the eye: the accessibility, relatively immune-privileged and tight blood-ocular barriers[3, 4]. Different AAV serotypes transduce different cell types and tissues in eye. For example, AAV2 and AAV5 vectors can transduce retinal pigment epithelial cells (RPE) and photoreceptors, but AAV1and AAV4 vectors exclusively transduce RPE cells[5]. The route of administration of AAV vectors also influence the tropism of AAV in the eye. Subretinal injection of AAV2 vectors results in transduction of RPE and photoreceptors, whereas intravitreal injection leads to ganglion cells transduction[4].

Although AAV is widely used as a gene delivery vehicle for ocular gene therapy, especially in retinal disease[3], the anterior segment of the eye, especially the trabecular meshwork (TM) cells cannot be efficiently transduced by AAV2, AAV3, AAV4[2, 6]. Borras et al revealed that host downregulation of DNA replication was one of the rate-limiting step of AAV transduction human trabecular meshwork cells[7]. Subsequently, scAAV2 which can bypass the rate-limiting step of second-strand synthesis was tested and shown to efficiently transduce the anterior segment cells of the eye in mouse, rat and rhesus[8, 9]. Moreover, scAAV2 capsid with tyrosine mutations delivered more GFP to cornea endothelia and TM than wild type scAAV2 via anterior chamber injection[10]. Unfortunately, the package genome capacity for scAAV is approximately 2.2kbs, which is limiting many applications due to the fact that the therapeutic cDNA or regulatory sequences (e.g. cell specific promoters) often exceeds this size[8]. Traditional ssAAV can package transgenes of slightly more than double this number of basepairs. For cornea, AAV8 was reported to be efficient in targeting corneal stroma by topical administration after removing the epithelium[11] and by intra-stroma injection in mice[12]. Human cornea explants were also transduced by AAV8, AAV8 and 9 chimeric capsid (8G9) by intra-stromal injection[12, 13].

Glaucoma is the second leading cause of blindness worldwide. It is characterized by the death of retinal ganglion cells (RGCs) and loss of vision. Targeting trabecular meshwork (TM) tissue, is one main avenue in the research of gene therapy for glaucoma[14]. In addition, efficient cornea transduction will advance the development of new therapies in inherited cornea dystrophies or systemic diseases involving the cornea, like mucopolysaccharidosis[13, 15].

In contrast to most AAVs, Anc80L65 is synthetic by design based on ancestral sequence reconstruction. Data from our group shows Anc80L65 can achieve fast onset and long-term stable expression in retinal cells, as well as liver and muscle, unlike many other AAVs[16]. Hair cells in the murine cochlea, known to be poorly permissive to most AAVs, can be transduced efficiently with Anc80L65 [17]. Building on these observations, we explored the tropism and transduction efficiency for cell targets in the anterior segment of the eye of Anc80L65 known to be refractory to transduction with ssAAV via single intracameral injection.

## Results

### Anc80L65, AAV2, AAV8 and scAAV2 vectors *in vitro* infectivity

**Fig 1:**
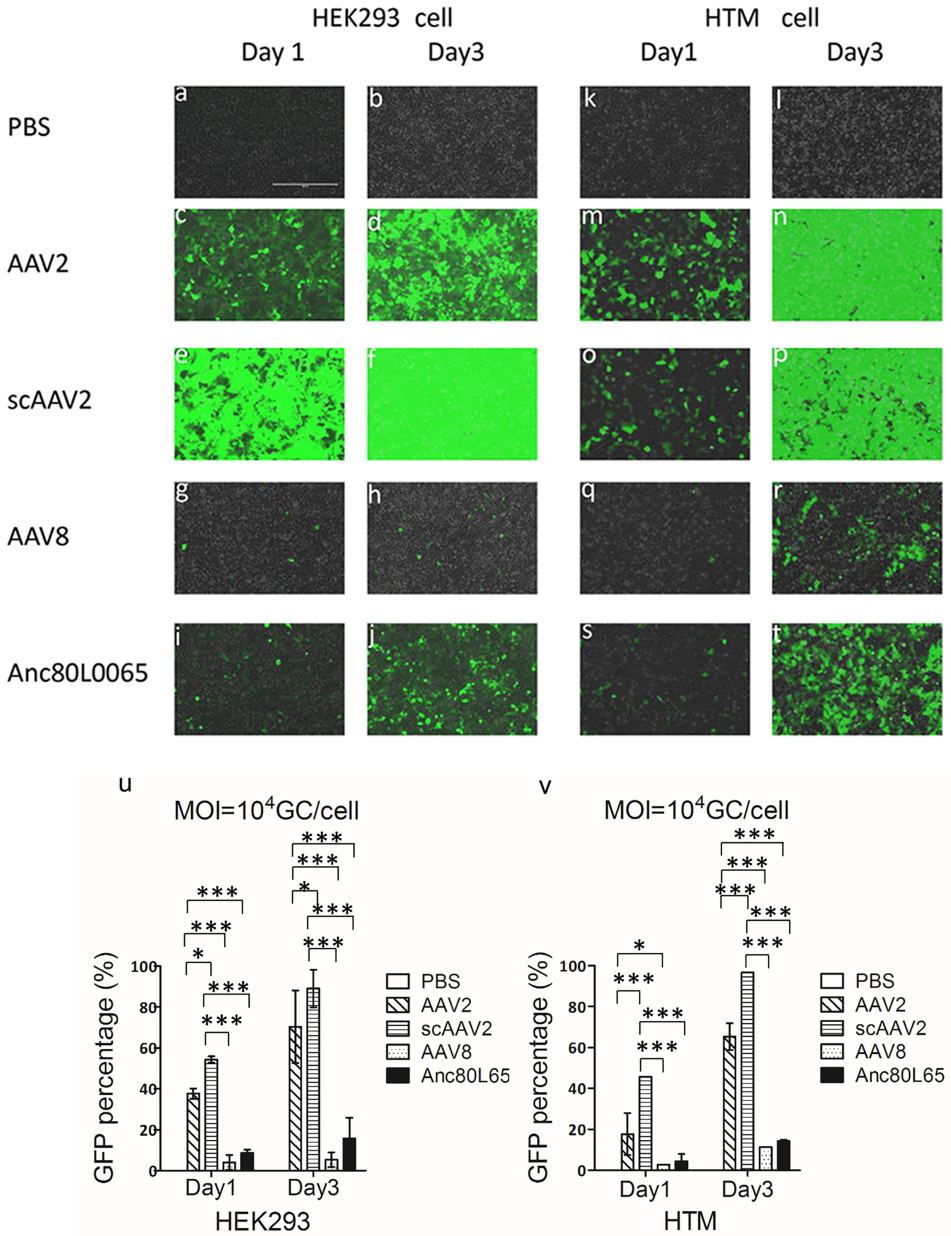
Representative images of GFP expression in the HEK293 and HTM cells infected with PBS, AAV2, scAAV2, AAV8 and Anc80L65 with a MOI of 1×10^4^ GC/cell (a-t). Images were taken under digital inverted microscope at 1 and 3 days after infection (10x magnification). The scale bar is 400 μm. GFP positive cell percentage of HEK293 and HTM cells 1 and 3 days after infection(u-v). Means and standard deviations of three independent experiments are shown (* = p<0.05, ** = p<0.01, *** = p<0.001).

We first evaluated infectivity of ssAAV2, 8, and Anc80L65 in Human embryo kidney 293 cells (HEK293) and Human trabecular meshwork cells (HTM) in comparison to scAAV2 controls. Transduction was qualitatively monitored for GFP transgene expression at several time points following infection. With a MOI of 1×10^3^ genome copies/cell (GC/cell), GFP was observed 1 day after infection with scAAV2 in HEK293 and HTM cells, expression increased at 3 days (S1 Fig) and was detected up to 5 days after infection (data not displayed). We also detected minimal expression of AAV2 at 1 day after infection in HEK293 and HTM cells (S1 Fig). Cells infected with AAV8 and Anc80L65 vectors did not show GFP expression (S1 Fig). However, with a MOI of 1×10^4^ GC/cell, GFP expression was detected 1 day after infection with AAV8 and Anc80L65 in HEK293 and HTM cells (Fig 1g, 1i, 1q and 1s) and increased at 3 day after infection (Fig 1h, 1j, 1r and 1t) and was detected 7 days after infection (data not displayed). We found higher GFP expression in HEK293 and HTM cells infected with scAAV2 and AAV2 (Fig 1c–1f and 1m–1p) compared to GFP expression in these cells with a MOI of 1×10^3^ GC/cell. The amount of GFP expressing in HEK293 and HTM cells at 1 and 3 days after transduction with a MOI of 1×10^4^ GC/cell in different serotypes was showed in Fig 1(Fig 1u and 1v). Using a MOI of 1×10^4^ GC/cell, scAAV2 lead to a 6.5-fold higher transduction rate (p<0.001) in HEK293 cells and a 10.5- fold higher transduction rate (p<0.001) than with Anc80L65 at 1 day after infection.

### Anc80L65-GFP vectors robustly transduce the cornea

**Fig 2:**
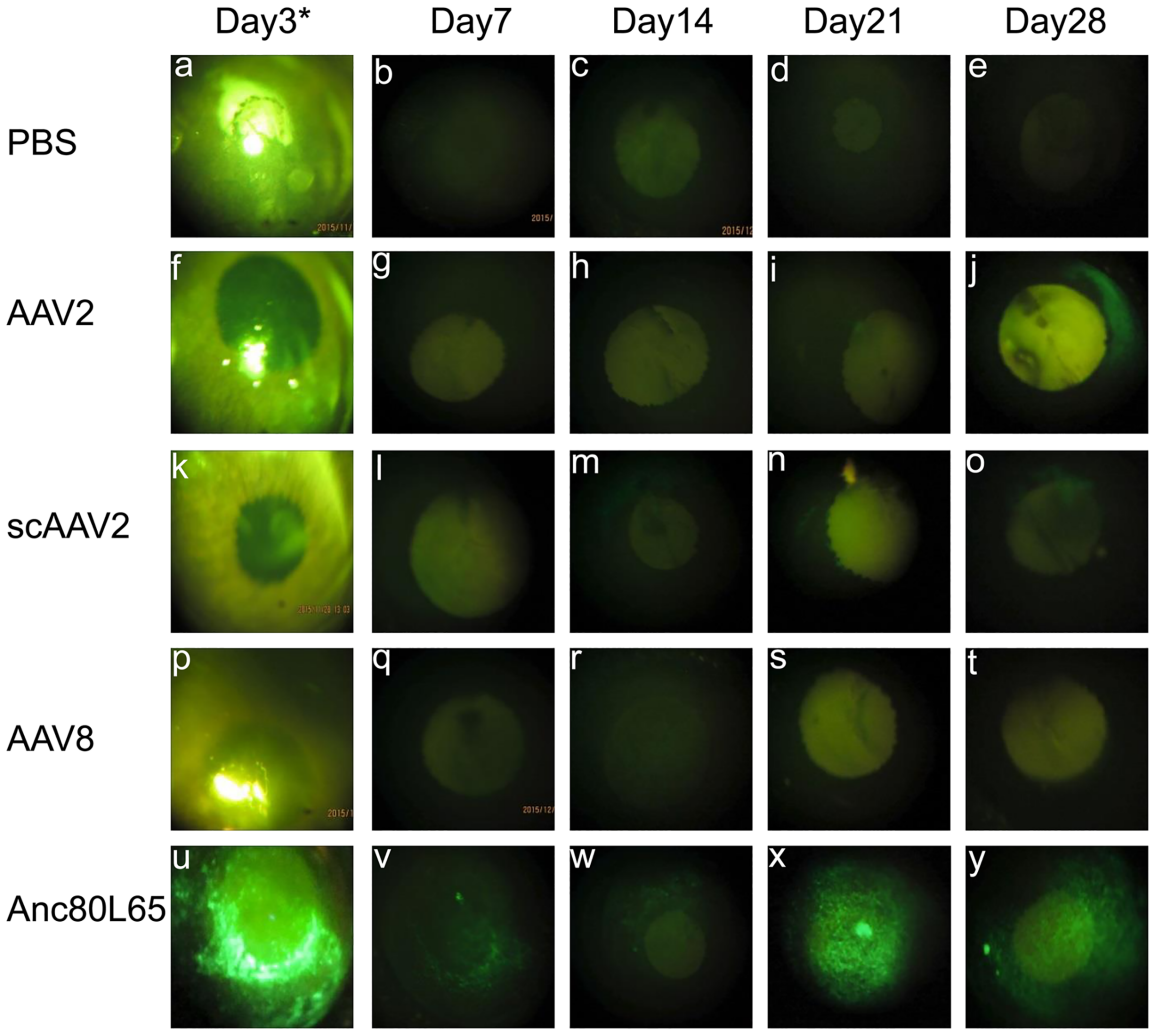
Representative images of GFP expression in the mouse cornea injected with different serotypes under stereoscopic microscope at day 3, 7, 14, 21 and 28 after injection. Images a-e indicates PBS group, f-j indicates AAV2 group, k-o indicates scAAV2 group, p-t indicates AAV8 group, u-y indicates Anc80L65 group. * indicates that images at day 3 post-injection taken under bright illumination.

Next, we aimed at evaluating Anc80L65 tropism for anterior segment targets *in vivo*. Mice were injected with different serotypes carrying GFP as a reporter transgene. Three days after the injection, we detected by stereomicroscopy bright spots in the cornea in AAV2, AAV8, scAAV2 and PBS groups, which were not positive for GFP expression, they were considered as light reflection under bright illumination (Fig 2). In order to remove the reflection, cornea images at 7, 14, 21 and 28 days post-injection were captured without bright illumination. By stereoscopic microscopy, GFP expression in the cornea was found in mice injected with Anc80L65 three days after injection (Fig 2u) and GFP signals increased gradually overtime until day 28 post-injection (Fig 2v–2y). We detected weak GFP signal for AAV2 at day 14 (Fig 2h) and scAAV2 at 7 day in the cornea (Fig 2l). The GFP expression with scAAV2 increase at day 14 and 21 under stereoscopic microscope (Fig 2m and 2n), but at day 28, the mouse cornea injected with AAV2 vectors showed stronger GFP expression than mice injected with scAAV2 (Fig 2j and 2o). For the AAV8 and PBS groups, we did not find any green fluorescence in the cornea after injection (Fig 2a–2e and 2p–2t).

**Fig 3:**
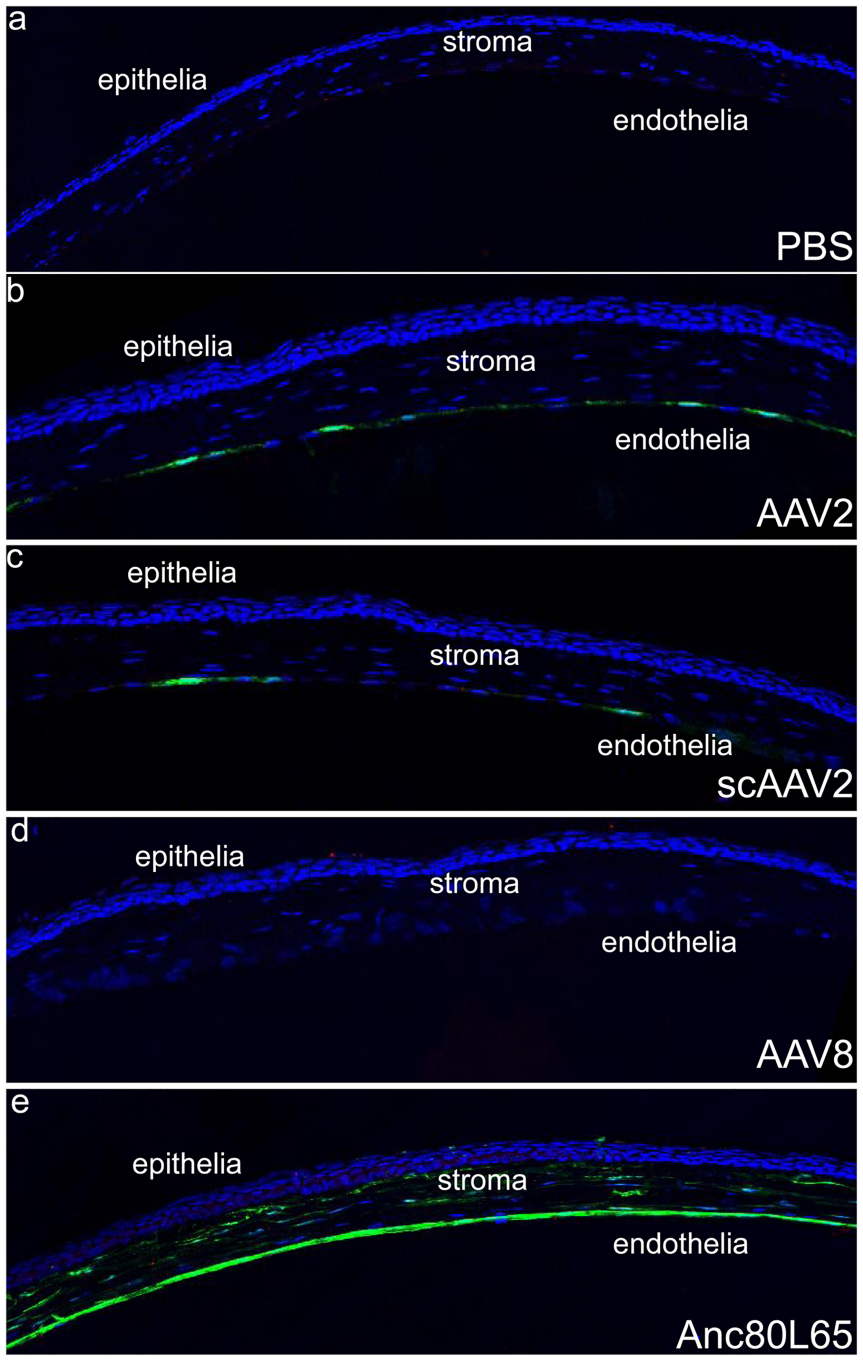
Representative images of GFP expression in the cornea stroma and endothelial cells of mice injected with different serotypes under confocal microscopy 28 days after injection. Nuclei were stained with DAPI (blue).

Histological analysis showed that eyes injected with Anc80L65 vectors 28 day after injection exhibit robust GFP expression in the corneal stroma and endothelial cells (Fig 3e). GFP expression was only detected in the cornea endothelial layer in mice injected with AAV2 and scAAV2(Fig 3b and 3c). Moreover, mice injected with AAV2 showed more GFP expression in the cornea endothelia than with scAAV2 vectors, which is consistent with the performance under stereoscopic microscopy. AAV8 and PBS groups showed no GFP expression under confocal microscope (Fig 3a and 3d).

### Anc80L65-GFP vectors target trabecular meshwork and ciliary body of mouse

**Fig 4:**
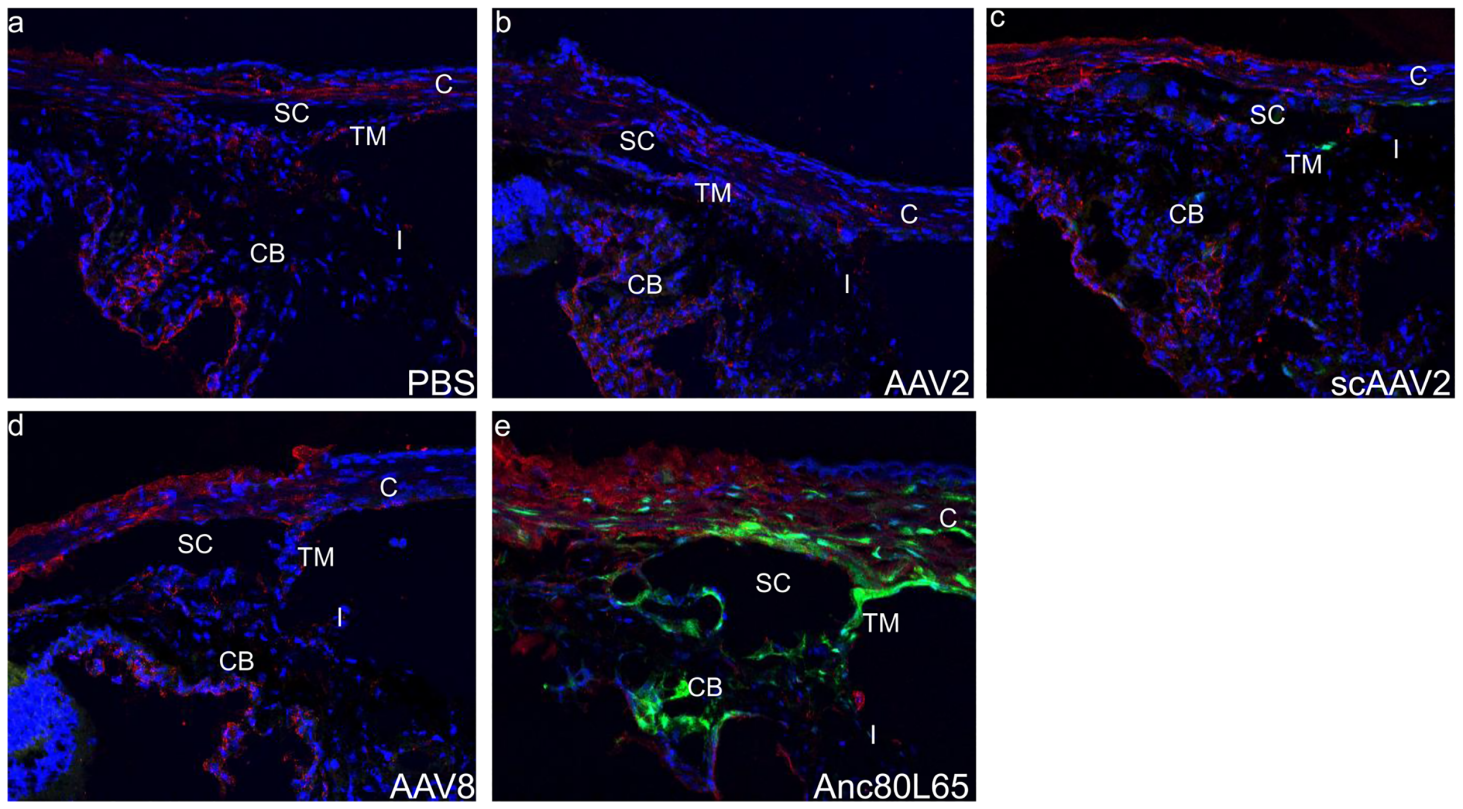
Representative images of GFP expression in the trabecular meshwork and ciliary body of mice injection with different serotypes under confocal microscopy 28 days after injection. The mouse anterior chamber angle tissue was stained with thrombospondin-1(red). Nuclei were stained with DAPI (blue). C indicates cornea, I indicates iris, CB indicates ciliary body, TM indicates trabecular meshwork, SC indicates schlemm’s canal.

Subsequently, we examined the GFP expression in the trabecular meshwork in all the groups injected in the previous experiments. Intense GFP expression was found in the trabecular meshwork, Schlemm’s canal and ciliary body of mice injected with Anc80L65 vector (Fig 4e). Mice injected with scAAV2 vectors showed lower GFP expression in the trabecular meshwork and ciliary body (Fig 4c). No GFP signal was found in the trabecular meshwork and ciliary body of mice injected with AAV2, AAV8 vector and control group (Fig 4a, 4b and 4d).

### Slit Lamp microscope examination of the anterior segment of mouse eye

3 days after the injection of viral vectors, no obvious leakage from the anterior chamber to the cornea in the puncture sites was found under the slit lamp microscope examination. A mild inflammation in the anterior chamber, associated with the injection procedure and independent of the treatment, was detected in 5 mice. It was completely resolved after 7 days.

### IOP measurement

**Fig 5:**
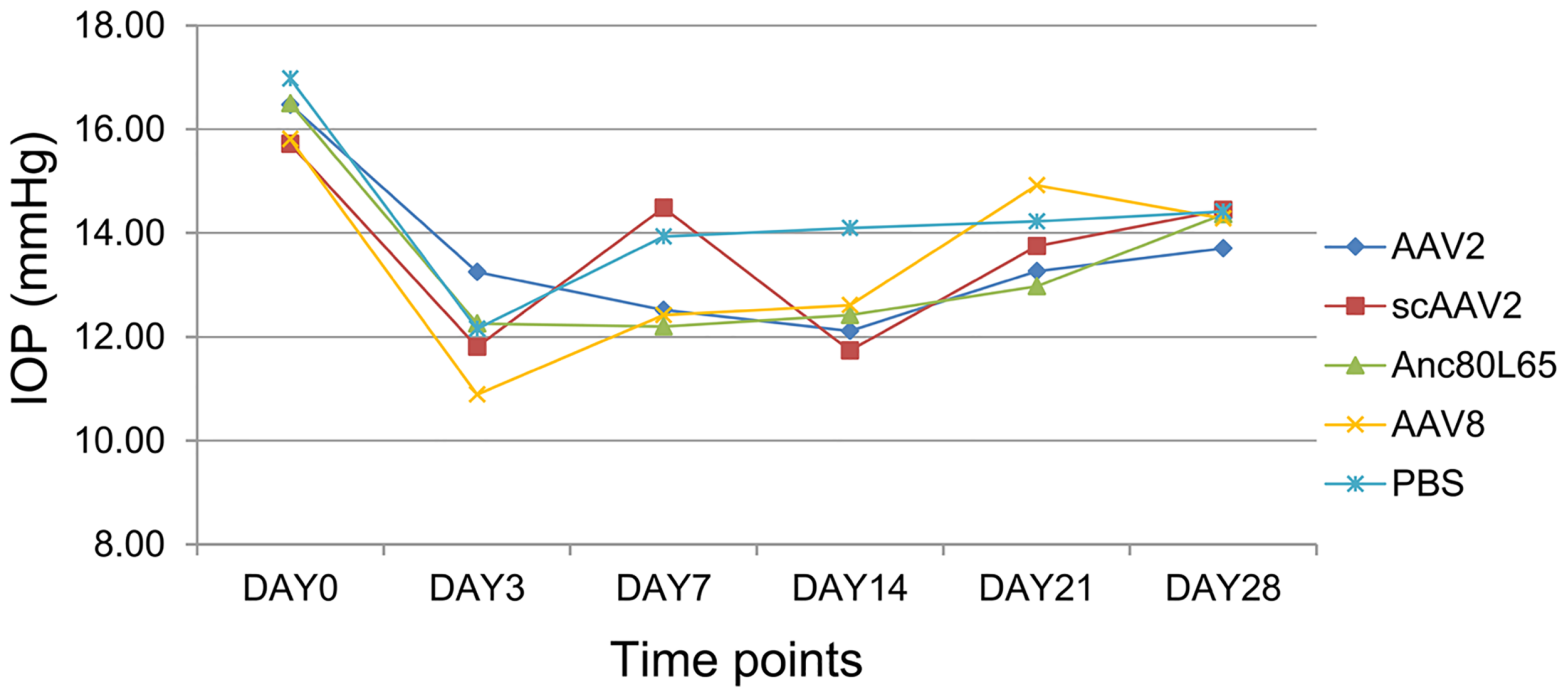
IOP changes of mice injected with PBS, AAV2, scAAV2, AAV8 and Anc80L65 before injection and at different time points after injection.

IOP measurement did not show changes in the intraocular pressure at different time points after injection compare with the pressure measurements before injection with different vectors (Fig 5). We detected slightly lower intraocular pressure at 1 day after the injection in all the groups as a consequence of the injection. Subsequently, the pressure went up gradually to normal levels in all the injected mice.

## Discussion

There is an unmet need for diseases of the anterior segment of the eye for which gene therapy approaches conceivable may be able to intervene in the disease process. First, glaucomatous diseases caused by obstruction of the outflow track of the eye are often due to defects in the TM. The TM is responsible for draining around 85% of the aqueous humor produced by ciliary body into scleral plexuses and general blood circulation. Therapeutic options in glaucoma are minimal, and often poor in long term outcome. Second, corneal disorders of various etiologies affecting many people yet have often a variety of therapeutic options including eye drops, other pharmacological treatments, and as a last resort, corneal transplantation. Compliance of the drug regimen, particularly in chronic corneal indications, and transplant rejection remain the primary obstacles to improve outcomes for corneal disease. Potential immediate opportunities for gene therapy in anterior segment ocular disease may lie in single gene disorders such as mutant myocilin (MYOC) -associated juvenile open angle glaucoma (JOAG) and corneal clouding in lysosomal storage disorders, like alpha-L-iduronidase (IDUA)-associated mucopolysaccharidosis type 1(MPS1).

Two of the primary interior segment targets of the eye, cornea and TM, appear refractory to transduction with ssAAV due to the apparent restriction of corneal endothelium and TM alike to perform AAV second strand synthesis. Previous reports describe that this limitation can be overcome by scAAV, enabling robust expression in TM and cornea following intracameral injection. The requirement to use scAAV however is known to significantly limit the genome cargo capacity from approximately 4.5kB for ssAAV to 2.2kB for scAAV. This restricted genome size prevents large transgene cassettes from being considered for gene transfer to the anterior segment, significantly impacting the number of opportunities for AAV gene transfer for anterior segment indications. For example, both MYOC and IDUA genes have longer cDNA than 2kB, which limit scAAV application as a vector in gene therapy of JOAG and MPS1.

Genome conversion appears to be a key restriction to corneal and TM transduction upon gene transfer. However, gene transfer can only be successfully accomplished by vectors that are able to enter a particular target cell. Different AAV serotypes have been described to indeed have altered tropism across tissues and cell targets, presumed to be due to sequence variation in the AAV capsid structure that allows it to interact distinctly with host co-factors relevant to AAV entry and post-entry steps in its path to the nucleus where second strand synthesis is believed to occur. Data to this effect has been available in both the cornea and the TM; scAAV2 capsid with tyrosine mutations are able to deliver more GFP to the cornea and TM than wild type scAAV2 and other capsid mutants via anterior chamber injection[10].

Multiple AAV vectors have been reported to transduce different corneal cells *in vitro*[18, 19], and *in vivo*[15, 20] in different species. AAV8 was reported to transduce human corneal fibroblasts *in vitro*[19] and the cornea stroma in the mouse *in vivo* by topical administration after removing the epithelium[11]. Intra-stromal injection of AAV8 expressing GFP showed fluorescence expression in the corneal stroma keratocytes of mice *in vivo* lasting for 17 months and human cornea explants *ex vivo*[12]. In our studies, we detected mild GFP expression in HEK293 and HTM cell infected with AAV8 under high MOI. However, we did not find any GFP expression in the mouse cornea after intracameral injection with AAV8. This evidences that route of administration has an effect on the tropism and transduction efficiency.

In addition, for AAV2 and scAAV2, Buie et al found GFP positive cornea endothelia in rats and monkey after a single intracameral injection of scAAV2[9]. In contrast, the same route of administration was used by Bogner et al., they showed that wild type scAAV2 could not transduced corneal endothelium in mice and rats, but corneal endothelium in both species was effectively transduced by scAAV2 capsid mutants of highly conserved surface-exposed tyrosine residue[10]. However, recently Gruenert et al. demonstrated both scAAV2 and AAV2 containing GFP showed transgene expression in human corneal endothelial cells (HCEC-12) over 28 days and in human corneal tissue ex vivo after 6 days[21], with high transduction by scAAV2. Our study showed similar results. We demonstrated that AAV2 and scAAV2 induced eGFP expression in corneal endothelial cells instead of cornea stroma after intracameral injection. Differences in the gene delivery route, the vector preparation, and dosage, could explain the differences between the results obtained by multiple groups. We also found that scAAV2 vector induced slight early onset of transgene expression at day 7 and higher transgene expression than AAV2 at day 14 after injection, but at 28 day after injection, mice injected with AAV2 vectors showed more transgene expression than mice injected with scAAV2. scAAV2 might induce early onset of transgene expression due to double-strand transgene which can contribute to active transcriptional gene form, but this advantage does not seem to exploit in later stage.

Here, we evaluated Anc80L65 in comparison with AAV2 and AAV8 in their ability to target TM and cornea using a single stranded AAV vector. Anc80L65, an AAV capsid that was synthetically designed in our previous work, demonstrates potent gene transfer *in vivo* in the murine retina and other tissues[16, 22]. Importantly however, it has been shown to target otherwise poorly permissive cell types such as cochlear outer hair cells[17]. Lastly, preliminary data illustrates across several tissue targets a remarkably fast kinetics in terms of onset of expression with Anc80L65, possibly allowing for increased amounts of genome template to be available during a critical stage of genome conversion. We hypothesized for these studies that Anc80L65 carries a tropism for TM and/or cornea following intracameral injection, and that it overcomes the need for scAAV genomes for functional transduction, possibly due to its fast kinetics of transduction. Indeed, the data presented here demonstrate that Anc80L65 can efficiently transduce the cornea and trabecular meshwork in mice showing a robust GFP expression in comparison to other AAVs 2 and 8. Surprisingly, single stranded Anc80L65 even exceeds in certain cases scAAV2 transduction which was carried forward in our studies as a positive control. Moreover, Anc80L65 leads to early onset of the transgene expression in murine cornea, in comparison to the other tested AAVs. Lastly, by immunofluorescence, we confirmed Anc80L65 transduced the corneal stroma and endothelial cells robustly by a single injection. This is the first report of early onset simultaneous transduction in cornea stroma and endothelium by AAV vectors.

In conclusion, our data demonstrates that Anc80L65 holds promise as a gene transfer vector for gene therapy approaches to therapeutic targets in the anterior segment of the eye. In particular, corneal endothelium and stroma in addition to the TM, are efficiently transduced with Anc80L65. Anc80L65 also overcomes the restriction of other AAVs at the stage of single stranded genome conversion in these target cells in that this AAV vector allows for potent transduction of cornea and TM in a ssAAV conformation. Our data therefore proposes Anc80L65 for further evaluation for their therapeutic application in corneal and glaucomatous diseases.

## Materials & methods

### Cell culture and infection assay

HTM cells were kindly provided by Dr. Vincent Raymond (Laboratory of Ocular Genetics and Genomics, Québec City, Canada)[23]. HTM and HEK293 cells were grown in Dulbecco’s modified Eagle’s medium (DMEM) with 4.5 g/L glucose, L-glutamine and sodium pyruvate, 10% fetal bovine serum, 100 U/mL penicillin, and 100μg/mL streptomycin sulfate and maintained in a humidified 5% CO_2_ environment at 37°C. All culture reagents were obtained from Mediatech Inc. (A Corning Subsidiary, Manassas, VA). Cells were plated in 6-well plates (Costar, Corning incorporated, NY) for cell infection (6×10^5^ cells/well) 24 hours prior to infection experiments and were incubated at 37°C until 100% confluent. The cells were then infected with different serotypes (dilutions prepared with culture media) at multiplicity of infection (MOI) of 1×10^3^ GC/cell and 1×10^4^ GC/cell. From day 1 to day 5 after infection, GFP expression levels in cells treated with different vectors were observed and photographed under digital inverted microscope with camera. GFP positive cell percentage at day 1 and 3 after infection was quantified by Countess II FL Automated Cell Counter with software version 1.0.238 (Thermo Fisher Scientific Inc., USA) under same fluorescence intensity. Three individual counts for each cell type under each MOI were taken under same gain. Statistical analysis was performed using GraphPad Prism (version 5.01). All values are expressed as mean ± SD. Significance was determined by two-way ANOVA analysis with Bonferroni post-tests correction. Significance was set at *p* ≤ 0.05.

### Experimental animals

C57Bl/6 male mice (6–8 weeks old) were purchased from Jackson Laboratory and kept at the Schepens Eye Research Institute (SERI) Animal Facility. This study was carried out in strict accordance with the recommendations in the Guide for the Care and Use of Laboratory Animals of the National Institutes of Health. All animal procedures were performed in accordance with protocols approved by approved by the Institutional Animal Care and Use committee (IACUC) at SERI. To ameliorate any pain or distress during ocular injections, the mice were anesthetized with intraperitoneal injection of ketamine/xylazine at a mixture of ketamine 100 mg/kg, xylazine 20 mg/kg. The mice were humanely euthanatized with carbon dioxide inhalation followed by cervical dislocation in accordance with SERI IACUC guidelines.

### AAV-vector construction and production

All AAVs serotypes used during this study contained the cytomegalovirus (CMV) promoter driving enhanced green fluorescent protein (GFP) reporter cDNA and Woodchuck Hepatitis Virus Posttranscriptional Regulatory Element (WPRE). AAV vector preparations were performed by triple plasmid transfection method according to methods described previously[24]. Briefly, large-scale polyethylenimine transfections of AAV cis, AAV trans, and adeno-virus helper plasmid were performed with near-confluent monolayers of HEK293 cells. Plasmids were transfected at a ratio of 2:1:1. The transfection and downstream purification process were performed following protocols described previously[24]. Viral vectors were resuspended in phosphate-buffered saline (PBS). DNase-I-resistant vector genomes copies were used to titrate AAV preparations by TaqMan qPCR amplification (Applied Bio-systems 7500, Life Technologies) with primers and probes detecting promoter, and transgene of the transgene cassette. The purity was evaluated by SDS-PAGE gel electrophoresis.

### Intracameral injections

Intracameral injections were performed at day 0 in C57Bl/6 mice (n = 6 per group for mice injected with Anc80L65, AAV2, AAV8 and scAAV2 vectors groups, n = 3 for the control group injected with PBS). Mice were anesthetized with a mixture of 100 mg/kg ketamine, 20 mg/kg xylazine and saline supplemented by topical anesthetic of 0.5% proparacaine hydrochloride (Bausch & Lomb, Tampa, FL). Under a surgical microscope (Leica Wild M-690 Mel53 Surgical Operating Microscope System, Leica Microsystems Inc., Switzerland), mice cornea was gently punctured with a 33-gauge needle (STERiJECT Premium disposable needles, TSK Laboratory, Japan) anterior of the iridocorneal angle and positive pressure was applied to create an air bubble with a Hamilton syringe attached to the 33-gauge needle. Then the needle was slowly removed to facilitate gentle movement of the air bubble towards the puncture site. A single injection of 1×10^9^ GC per eye was injected in each animal (final volume of 1μl), PBS as a control. Viral vectors or PBS was slowly injected via the same puncture site with a glass micropipette connected to a Hamilton syringe by polyethylene tubing. The glass micropipette was held in position for 1 minute, after the micropipette was removed, the air bubble was kept at the puncture site to prevent reflux of vector solution or PBS control. Neomycin and polymyxin B sulfates and bacitracin zinc ophthalmic ointment (Bausch & Lomb, Tampa, FL) were applied to the eye after the procedure.

### Slit lamp microscope examination, GFP detection with stereoscopic microscopy and IOP measurement

At day 3, 7, 14, 21 and 28 after injection, mice were anesthetized by an intraperitoneal injection of 100 mg/kg ketamine and 20 mg/kg xylazine. Cornea puncture healing and anterior chamber reaction in the mice eyes were observed by SL-D7 digital slit lamp microscope (Topcon Corporation, Tokyo, Japan). Cornea images with GFP expression for each animal were captured under stereoscopic zoom microscope (Nikon, SMZ 800, Japan) with digital compact camera (IXUS 300 HS, Canon, Japan), image in Figs 2 and 3 are representative images of each group. Cornea images for all groups at day 3 were taken under bright illumination and all other images were taken under dark illumination. IOP was measured with a rebound tonometer (ICare Tonolab, Espoo, Finland) at day0 before injection and day 3, 7, 14, 21 and 28 after injection. The instrument took five individual measurements and gave the mean as one reading. Five such readings were taken from each eye, and the average was calculated. Measurements were conducted at the same time in the morning for all groups.

### Tissue harvest and immunohistochemistry

At day 28 after injection, mice were euthanized by carbon dioxide, followed by cervical dislocation based on procedures approved by IACUC of SERI. Mice eyes were fixed in in 4% paraformaldehyde at 4°C for 30 minutes to 1 hour. After rinsing, the whole eyeball was immersed in OCT compound (Tissue-Tek^®^ OCT 4583: Sakura Finetek USA, Inc., Torrance, CA) and frozen quickly in a bath of liquid nitrogen and stored at -80°C until sectioning. The frozen eyeball was cross-sectioned at 14μm thickness using Leica CM1950 cryostat (Leica, IL, USA). The transverse section was incubated in blocking solution (5% normal goat serum, 1% BSA and 0.1% Triton X-100 in PBS) for 1 hour at room temperature. Afterwards, a primary antibody against thrombospondin-1 (mouse anti-TSP-1, clone A6.1, 1:100, Thermo Fisher Scientific Inc., USA) was applied at 4°C overnight. TSP-1, a matricellular protein, is used as a marker to distinguish TM structures from others in the anterior chamber angle[10]. Sections were washed in 1xPBS for 3x10 min, followed by an Alexa Fluor 555-conjugated secondary antibody (goat anti-mouse, 1:500, Thermo Fisher Scientific Inc., USA) for 2 hours at room temperature. Finally, sections were incubated with the nuclear dye 4', 6-diamidino-2-phenylindole (DAPI) (1:1000, Life Technologies, Grand Island, NY) for 30 minutes at room temperature, washed in 1XPBS, and mounted in Dako fluorescent mounting medium (Dako North America, Inc., CA, USA). The slides were analyzed and imaged with a 20X glycerol immersion objective of Leica confocal laser scanning microscope (TCS SP5 confocal system, Leica Microsystems, Buffalo Grove, IL). Samples from different animals in each group were imaged and processed in the same manner to ensure the consistency between the all the groups.

## Supporting information

S1 Fig Representative images of GFP expression in the HEK293 and HTM cells infected with a MOI of 1×10^3^ GC/cell with different serotypes (a-t). Images were taken under digital inverted microscope at 1 and 3 days after infection (10x magnification). The scale bar is 400 μm. GFP positive cell percentage of HEK293 and HTM cells 1 and 3 days after infection(u-v). Means and standard deviations of three independent experiments are shown (* = p<0.05, ** = p<0.01, *** = p<0.001).
(TIF)
