# Supplementary figures and images for: Single stranded adeno-associated virus achieves efficient gene transfer to anterior segment in the mouse eye
Source: PLoS One. 2017 Aug 1;12(8):e0182473. doi: 10.1371/journal.pone.0182473 (PMC5538712; doi:10.1371/journal.pone.0182473)

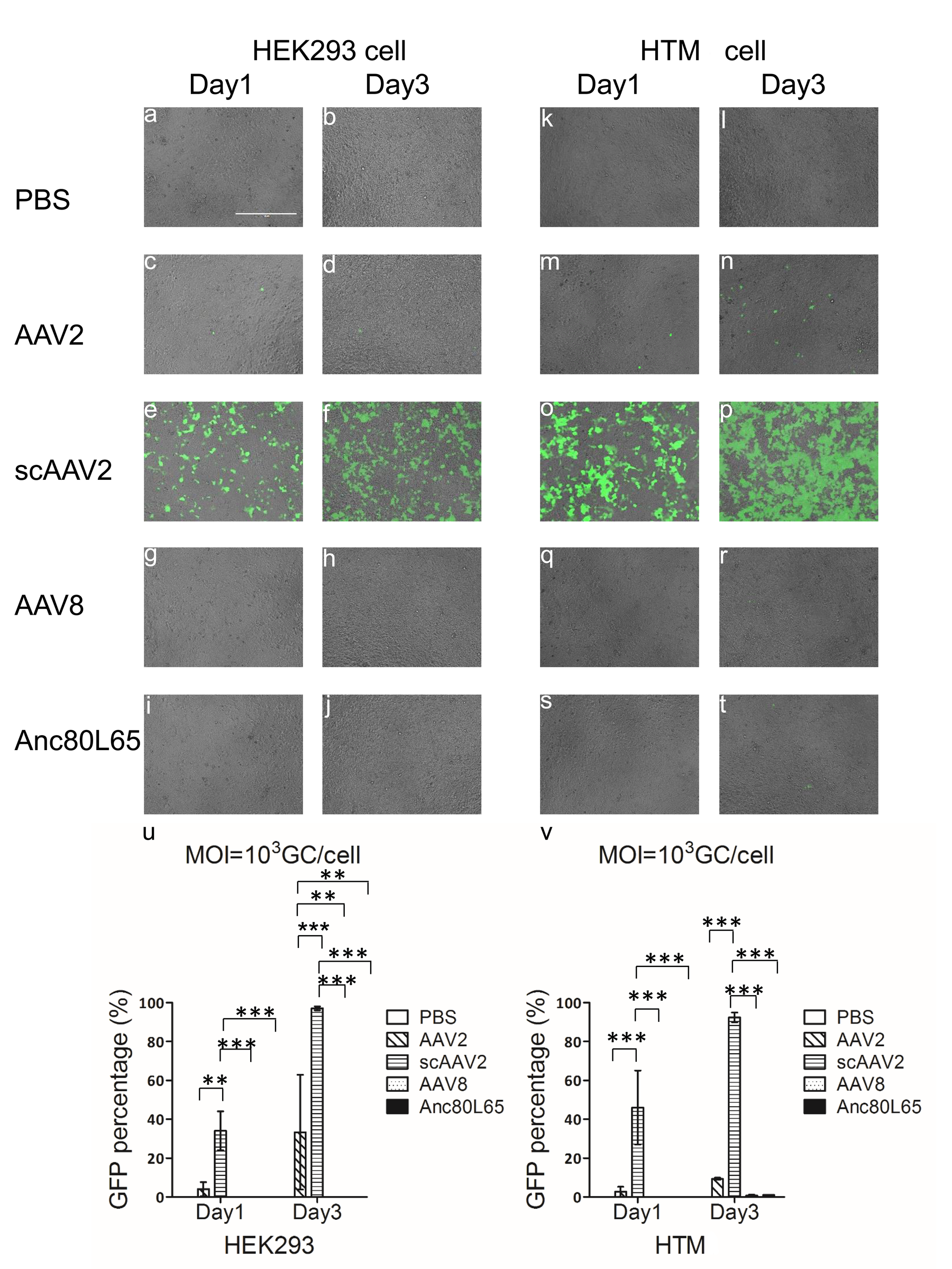

Supplement: S1 Fig — Images were taken under digital inverted microscope at 1 and 3 days after infection (10x magnification). The scale bar is 400 μm. GFP positive cell percentage of HEK293 and HTM cells 1 and 3 days after infection(u-v). Means and standard deviations of three independent experiments are shown (* = p<0.05, ** = p<0.01, *** = p<0.001). (TIF) [file pone.0182473.s001.tif]
